# Insanity in Relation to Sex and Age

**Published:** 1915-02

**Authors:** 


**Insanity in Relation to Sex and Age.** The report on the insane in the United States, prepared by Dr. Joseph A. Hill and recently issued by William J. Harris, director of the census, Department of Commerce, indicates that there is more insanity among men than among women. January 1, 1910, there were 98,695 males in institutions for the insane, as compared with 80,069 females, and during the year 1910, 34,116 males were admitted, as compared with 26,623 females. There were 208 male inmates of insane asylums to every 100,000 males in the total population, while the corresponding ratio of female inmates to total female population was 199.6 to 100,000. The males admitted during the year 1910 represented a ratio of 72.1, the females, a ratio of 59.7. If the cases of alcoholic psychosis and general paralysis are deducted, the disparity between the sexes practically disappears. There are left of the total admissions 25,708 males as compared with 24,611 females, a slight excess of males, but no greater than naturally would result from the fact that there are more males than females in the general population.

The table following shows the number of admissions to hospitals for the insane in 1910:

| | Males. | Females. |
|---|---|---|
| Having general Paralysis | 3,041 | 1,806 |
| Having Alcoholic Psychosis | 5,220 | 902 |
| Having both diseases | 147 | 54 |
| All other cases | 25,708 | 24,611 |
| Total number admitted | 34,116 | 26,653 |

Ratio of admissions to hospitals for the insane per 100,000 population of the same sex and age.

| | All causes. | | Cases of general paralysis and alcoholic psychosis. | | Other cases. | |
|---|---|---|---|---|---|---|
| | M. | F. | M. | F. | M. | F. |
| All ages | 72.1 | 59.7 | 17.7 | 4.1 | 54.4 | 55.6 |
| Under 15 years of age | 1.2 | 1.0 | 0.1 | 0.1 | 1.1 | 0.9 |
| 15 to 19 years of age | 32.5 | 23.5 | 1.1 | 0.7 | 31.3 | 22.9 |
| 20 to 24 years of age | 70.6 | 55.1 | 5.9 | 2.1 | 64.8 | 53.0 |
| 25 to 29 years of age | 92.1 | 79.2 | 16.0 | 3.9 | 76.1 | 15.2 |
| 30 to 34 years of age | 109.9 | 98.8 | 29.8 | 6.7 | 80.0 | 92.2 |
| 35 to 39 years of age | 121.5 | 112.4 | 41.9 | 9.5 | 79.6 | 102.9 |
| 40 to 44 years of age | 129.8 | 115.2 | 48.6 | 12.2 | 81.3 | 102.9 |
| 45 to 49 years of age | 133.0 | 120.5 | 47.5 | 11.1 | 85.4 | 109.4 |
| 50 to 54 years of age | 128.5 | 120.9 | 42.9 | 9.7 | 85.6 | 111.2 |
| 55 to 59 years of age | 132.7 | 107.3 | 39.1 | 8.1 | 93.6 | 99.2 |
| 60 to 64 years of age | 143.2 | 108.6 | 30.4 | 7.3 | 112.8 | 101.3 |
| 65 to 69 years of age | 145.3 | 114.8 | 24.4 | 7.2 | 120.8 | 107.5 |
| 70 to 74 years of age | 177.0 | 141.6 | 15.0 | 5.4 | 162.0 | 136.2 |
| 75 to 79 years of age | 204.1 | 150.0 | 18.7 | 7.7 | 185.3 | 142.3 |
| 80 years and over | 224.0 | 192.7 | 14.8 | 5.8 | 209.1 | 187.0 |

